# Prevalence of anaemia and associated factors among people with pulmonary tuberculosis in Uganda

**DOI:** 10.1017/S0950268822000103

**Published:** 2022-01-13

**Authors:** Joseph Baruch Baluku, Ernest Mayinja, Pallen Mugabe, Kauthrah Ntabadde, Ronald Olum, Felix Bongomin

**Affiliations:** 1 Division of pulmonology, Kiruddu National Referral Hospital, Kampala, Uganda; 2 Makerere University Lung Institute, Kampala, Uganda; 3 Directorate of programs, Mildmay Uganda, Wakiso, Uganda; 4 MRC/UVRI & LSHTM Uganda Research Unit, Entebbe, Uganda; 5 School of Medicine, Makerere University College of Health Sciences, Kampala, Uganda; 6 Department of Medical Microbiology & Immunology, Faculty of Medicine, Gulu University, Gulu, Uganda

**Keywords:** Anaemia, immunity, nutrition, severity, tuberculosis, Uganda

## Abstract

Anaemia predicts delayed sputum conversion and mortality in tuberculosis (TB). We determined the prevalence and factors associated with anaemia among people with TB at the National Tuberculosis Treatment Centre in Uganda. People with bacteriologically confirmed TB were consecutively enrolled in a cross-sectional study between August 2017 and March 2018. Blood samples were tested for a full blood hemogram, HIV infection, and CD4+ and CD8+ T-cell counts. Anaemia was defined as a haemoglobin level of <13.0 grams per decilitre (g/dl) for males and <12.0 g/dl for females. Of 358 participants, 210 (58.7%, 95% confidence interval (CI) 53.4–63.8) had anaemia. Anaemia was associated with night sweats, a longer duration of fever, low body mass index (BMI), hyperthermia, high sputum bacillary loads, HIV co-infection, and low CD4 and CD8 counts at bivariate analysis. Factors associated with anaemia at multivariable analysis were low BMI (odds ratio (OR) 2.93, 95% CI 1.70–5.05, *P* < 0.001), low CD4:CD8 ratio (OR 2.54, 95% CI 1.07–6.04, *P* = 0.035) and microcytosis (OR 4.23, 95% CI 2.17–8.25, *P* < 0.001). Anaemia may be associated with the features of severe TB disease and should be considered in TB severity scores.

## Introduction

Until 2020, tuberculosis (TB) was the leading cause of death from a single infectious agent and claimed the lives of at least 1.4 million individuals in 2019 [1]. In sub-Saharan Africa, the treatment success rate in bacteriologically confirmed TB patients was recently reported to be only 76% against a global target of 90% [2]. There is the need to identify and modify risk factors for poor treatment outcomes to realise the aims of the ‘End TB Strategy’ that espouses management of comorbidities as a key component of integrated patient care and prevention [3].

Anaemia is associated with a fourfold risk for TB infection and a dose-dependent relationship between anaemia severity and TB risk has been demonstrated in a recent systematic review and meta-analysis [4]. Further, anaemia is common among people with TB with an estimated prevalence of about 62%, and 36%, 31% and 12% of patients are reported to have mild, moderate and severe anaemia, respectively [5]. TB-associated anaemia has multiple causes, including suppression of erythropoiesis by inflammatory markers as well as nutritional deficiency [6]. The high prevalence of anaemia in TB is concerning because of its association with delayed sputum conversion [7], severe forms of TB (such as meningitis and disseminated disease) [8], TB-related mortality and TB recurrence [9]. It is, therefore, imperative to screen for anaemia among people with TB and institute timely interventions.

Uganda is an HIV/TB high-burdened country which notified almost 66 000 TB cases in 2019 and registered a TB treatment success rate of 74% for the 2017 cohort [1]. Malnutrition accounts for a higher number of the estimated incident TB cases (42 000) than HIV infection (33 000) [1]. However, the burden of anaemia in TB and its associations are not well established in Uganda. A study conducted more than 20 years ago at the National Tuberculosis Treatment Center (NTTC) reported anaemia among 63% and 43% of men with TB with and without HIV co-infection, respectively, while 86% and 68% of women with TB with and without HIV co-infection had anaemia, respectively [10]. Additionally, TB was the commonest diagnosis (diagnosed among 22%) in people with anaemia at the emergency unit of a national referral hospital [11]. We have recently shown that HIV-negative TB patients with anaemia were more likely to have low CD4 T-lymphocytes at the NTTC [12]. These studies suggest that the prevalence of anaemia among TB patients in Uganda may be high and could be associated with impaired immune responses and severe TB disease. The objective of this study was to determine the prevalence of anaemia and associated factors among people with bacteriologically confirmed TB at the NTTC in Uganda.

## Materials and methods

### Study design, population and setting

Using a cross-sectional study design, people presenting to the NTTC at Mulago National Referral Hospital (MNRH) in Uganda were enrolled consecutively between August 2017 and March 2018. The NTTC is an urban tertiary care facility for adult in-patient and outpatient TB care located in Kampala, the capital city of Uganda. It is a centre of excellence for TB care where both drug-sensitive and drug-resistant TB cases are managed. Less than 30% of TB cases managed at the facility are referrals from peripheral facilities. Eligible people were adults (≥18 years) who had pulmonary TB that was bacteriologically confirmed by sputum smear microscopy (Auramine staining), Xpert MTB/RIF assay and/or sputum mycobacterial culture (Löwenstein–Jensen medium). People who had received TB therapy for at least 2 weeks were excluded as TB therapy beyond 2 weeks alters clinical variables: mycobacterial burden, symptoms and haemoglobin levels [13, 14]. The findings of the primary study have been published elsewhere [15]. In this secondary analysis, we included all people in the primary database who had haemoglobin measurement performed. MNRH is a tertiary health care facility located in Kampala, the capital city of Uganda.

### Study definitions and measurements

Details of the study measurements are provided elsewhere [15]. Briefly, a study questionnaire that sought for demographic data, medical history and symptoms was administered by trained research assistants. The participants' weight and height were measured using a weighing scale (Seca 760^®^) and stadiometer (Seca 213^®^), respectively, and the body mass index (BMI) was computed using the formula: BMI = (weight in kilograms)/(height in centimetres)^2^. BMI was graded as: underweight (<18.5 kg/m^2^), normal (18.5–24.9 kg/m^2^) and overweight (≥25.0 kg/m^2^) [16]. An axillary temperature was measured using a digital thermometer and graded as: hypothermia (<35.5 °C), normal (35.5–37.4 °C) and hyperthermia (≥37.5 °C) [17]. Blood samples were drawn by a study nurse and were evaluated for HIV infection using immunochromatographic rapid tests according to the national guidelines [18]. Malaria infection was confirmed using thick blood smears and/or rapid diagnostic antigen tests (SD BIOLINE Malaria Ag P.f/Pan). A full hemogram was performed at MNRH haematology laboratory using an automated haemonalyser (Sysmex^®^ Automated haematology analyser XN series – XN 1000) following standard procedures. Anaemia was defined as a haemoglobin level of <13.0 grams per decilitre (g/dl) for males and <12.0 g/dl for females and graded as mild (11.0–12.9 g/dl for men and 11.0–11.9 g/dl for females), moderate (8.0–10.9 g/dl for both sexes) and severe (<8.0 g/dl for both sexes) [19]. Anaemia was classified as hypochromic if the mean corpuscular haemoglobin (MCH) was <24 picograms (pg) [20]. Microcytosis was defined as a mean corpuscular volume (MCV) of <76 femtolitres while macrocytosis was an MCV of >96 fl [20]. The MCH of <24 pg and MCH of <76 are considered appropriate cut-offs in screening for iron deficiency anaemia [20]. The CD4 and CD8 T-cell counts and the CD4:CD8 ratio were measured by flow cytometry (BD FACSCalibur™). Sputum bacillary load was graded as very low (Xpert MTB/RIF cycle threshold (Ct) >28 or 1–9 acid-fast bacilli (AFBs)/100 fields on smear microscopy), low (Ct 22–28 or 1–9 AFBs/10 fields), medium (Ct 16–22 or 1–10 AFBs/field) and very high (Ct <16 or >10 AFBs/field) [21, 22].

### Statistical analysis

Data were analysed using Stata 14 (StataCorp, College Station, TX, USA). Categorical variables were compared among people with and without anaemia using Pearson's *χ*^2^ or Fischer's exact tests as appropriate. Continuous variables were compared using the Mood's median test. Variables with *P* < 0.2 in bivariate analysis were entered into multivariate logistic regression model. We performed multivariable logistic regression analysis to determine factors that are independently associated with anaemia. Antiretroviral therapy (ART) use, and duration of weight loss and fever were dropped during model building to achieve a parsimonious model. Statistical significance was set, *a priori*, at *P* < 0.05 at the 95% confidence interval.

### Ethical approval and consent to participate

In the primary study, participants provided written informed consent before study procedures. The study was approved by the School of Medicine Research and Ethics Committee of Makerere University College of Health Sciences (REC REF 2017-087). The authors assert that all procedures contributing to this work comply with the ethical standards of the relevant national and institutional committees on human experimentation and with the Helsinki Declaration of 1975, as revised in 2008.

## Results

Of the 363 patients in the primary database, 358 (98.6%) had a haemoglobin measurement and were included in this analysis.

### Characteristics of TB patients with and without anaemia

Of 358 people with TB, 220 (61.5%) were males, 140 (39.1%) were aged 25–34 years, and 127 (35.5%) were HIV co-infected. Anaemia was prevalent among 210 (58.7%, 95% CI 53.4–63.8). As shown in Table 1, a higher proportion of patients with anaemia were cigarette smokers (21.6% *vs.* 33.8%, *P* = 0.012), HIV-positive (24.0% *vs.* 43.3%, *P* < 0.001) and reported night sweats (63.5% *vs.* 73.8%, *P* = 0.037), had hyperthermia (8.2% *vs.* 16.7%, *P* = 0.010) and a longer duration of fever (median days (IQR)) (14 (7–30) *vs.* 21 (14–52.2), *P* = 0.040). They also had very high sputum bacillary load (22.5% *vs.* 33.3%, *P* = 0.020) but lower median (IQR) BMI (19.5 (17.7–21.4) *vs.* 17.6 (16.0–19.7) kg/m^2^, *P* < 0.001), CD4 (564 (380–794) *vs.* 344 (146–613) cell/mm^3^, *P* < 0.001) and CD8 (460.5 (308.5–679.5) *vs.* 385 (225–585) cell/mm^3^, *P* = 0.040) T-cell counts, MCV (80.7 (73.8–88.9) *vs.* 76.3 (70.0–86.6) fl, *P* = 0.010), and MCH (27.4 (24.9–29.4) *vs.* 24.9 (22.6–27.5) pg, *P* < 0.001). However, more people without anaemia reported weight loss (32.4% *vs.* 18.1%, *P* = 0.002).

**Table 1.** Characteristics of TB patients with and without anaemia in Uganda

Characteristic	Total (*N* = 358)	No anaemia (*N* = 148)	Anaemia (*N* = 210)	*P* value
Sex				
Male	220 (61.5%)	92 (62.2%)	128 (61.0%)	0.820
Female	138 (38.5%)	56 (37.8%)	82 (39.0%)	
Age (years)				
15–24	85 (23.7%)	37 (25.0%)	48 (22.9%)	0.900
25–34	140 (39.1%)	57 (38.5%)	83 (39.5%)	
≥35	133 (37.2%)	54 (36.5%)	79 (37.6%)	
Level of education				
None/primary	159 (44.4%)	57 (38.5%)	102 (48.6%)	0.059
Secondary/tertiary	199 (55.6%)	91 (61.5%)	108 (51.4%)	
History of tobacco use				
Never smoked	255 (71.2%)	116 (78.4%)	139 (66.2%)	0.012
Ever smoked	103 (28.8%)	32 (21.6%)	71 (33.8%)	
History of alcohol use				
Never used	175 (48.9%)	76 (51.4%)	99 (47.1%)	0.430
Ever used	183 (51.1%)	72 (48.6%)	111 (52.9%)	
Type of residence				
Rural	116 (32.4%)	56 (37.8%)	60 (28.6%)	0.065
Urban	242 (67.6%)	92 (62.2%)	150 (71.4%)	
HIV test status				
Negative	231 (64.5%)	112 (75.7%)	119 (56.7%)	<0.001
Positive	127 (35.5%)	36 (24.3%)	91 (43.3%)	
Antiretroviral therapy (*n* = 127)				
No	65 (51.2%)	13 (36.1%)	52 (57.1%)	0.033
Yes	62 (48.8%)	23 (63.9%)	39 (42.9%)	
Cotrimoxazole use (*n* = 127)				
No	31 (24.4%)	7 (19.4%)	24 (26.4%)	0.413
Yes	96 (75.6%)	29 (80.6%)	67 (73.6%)	
Rifampicin resistance (*n* = 299)				
No	242 (80.9%)	99 (78.6%)	143 (82.7%)	0.370
Yes	57 (19.1%)	27 (21.4%)	30 (17.3%)	
Malaria positivity				
Positive	8 (2.2%)	3 (2.0%)	5 (2.4%)	0.820
Negative	350 (97.8%)	145 (98.0%)	205 (97.6%)	
Previous TB treatment				
No	304 (84.9%)	124 (83.8%)	180 (85.7%)	0.620
Yes	54 (15.1%)	24 (16.2%)	30 (14.3%)	
Cough				
Yes	352 (98.3%)	147 (99.3%)	205 (97.6%)	0.220
No	6 (1.7%)	1 (0.7%)	5 (2.4%)	
Cough duration in days (median (IQR))	60 (30–90)	30 (21–90)	60 (30–90)	0.210
Night sweats				
Yes	249 (69.6%)	94 (63.5%)	155 (73.8%)	0.037
No	109 (30.4%)	54 (36.5%)	55 (26.2%)	
Night sweats duration in days (median (IQR))	30 (14–60)	21 (10–30)	30 (14–60)	0.220
Anorexia				
Yes	264 (73.7%)	110 (74.3%)	154 (73.3%)	0.830
No	94 (26.3%)	38 (25.7%)	56 (26.7%)	
Anorexia duration in days (median (IQR))	14 (7–30)	14 (4–30)	14 (7–30)	0.600
Weight loss				
Yes	86 (24.0%)	48 (32.4%)	38 (18.1%)	0.002
No	272 (76.0%)	100 (67.6%)	172 (81.9%)	
Weight loss duration in days (median (IQR))	30 (14–60)	30 (14–45)	30 (21–60)	0.110
Fever				
Yes	142 (39.7%)	56 (37.8%)	86 (41.0%)	0.550
No	216 (60.3%)	92 (62.2%)	124 (59.0%)	
Fever duration in days (median (IQR))	14 (7–30)	14 (7–30)	21 (14−52.5)	0.040
Bacillary load (*n* = 346)				
Very low	46 (13.3%)	24 (16.9%)	22 (10.8%)	0.020
Low	80 (23.1%)	41 (28.9%)	39 (19.1%)	
Medium	120 (34.7%)	45 (31.7%)	75 (36.8%)	
Very high	100 (28.9%)	32 (22.5%)	68 (33.3%)	
Body mass index (BMI) (*n* = 354)				
Underweight	186 (52.5%)	52 (35.1%)	134 (63.8%)	<0.001
Normal	157 (44.4%)	87 (58.8%)	70 (33.3%)	
Overweight	11 (3.1%)	9 (6.1%)	2 (2.9%)	
Temperature (*n* = 356)				
Hypothermia	117 (32.9%)	59 (40.4)	58 (27.6)	0.010
Normal	192 (53.9%)	75 (51.4)	117 (55.7)	
Hyperthermic	47 (13.2%)	12 (8.2)	35 (16.7)	
CD4:CD8 ratio^a^(*n* = 356)				
<0.52	102 (28.7%)	21 (14.3%)	81 (38.8%)	<0.001
0.52–4.1	254 (71.3%)	126 (85.7%)	128 (61.2%)	
CD4/CD8 ratio (median (IQR))		1.39 (0.81–1.93)	1.06 (0.29–1.82)	0.070

a Cut-offs are for normal adult Ugandans [47].

### Severity of anaemia among people with TB in Uganda

Of the 210 people with anaemia, 85 (40.5%, 95% CI 33.8–47.4) had mild, 101 (48.1%, 95% CI 41.2–55.1) had moderate and 24 (11.4%, 95% CI 7.5–16.5) had severe anaemia. As shown in Table 2, most people with severe anaemia were HIV-positive (75.0%) compared to those with moderate (48.5%) and mild (28.2%) anaemia (*P* < 0.001). Similarly, most people with severe anaemia had low CD4 T-cell counts (83.3%) and CD4:CD8 ratio (79.2%) than those with moderate (67.3% and 43.6%) and mild anaemia (40.0% and 22.4%), respectively, *P* < 0.001.

**Table 2.** Characteristics of TB patients with mild, moderate and severe anaemia

Characteristic	Severe anaemia (*n* = 24)	Moderate anaemia (*n* = 101)	Mild anaemia (*n* = 85)	*P* value
Education				
Secondary/tertiary	12 (50.0%)	53 (52.5%)	43 (50.6%)	0.960
None/primary	12 (50.0%)	48 (47.5%)	42 (49.4%)	
History of tobacco use				
Never smoked	15 (62.5%)	74 (73.3%)	50 (58.8%)	0.110
Ever smoked	9 (37.5%)	27 (26.7%)	35 (41.2%)	
Residence				
Urban	17 (70.8%)	71 (70.3%)	62 (72.9%)	0.920
Rural	7 (29.2%)	30 (29.7%)	23 (27.1%)	
HIV status				
Negative	6 (25.0%)	52 (51.5%)	61 (71.8%)	<0.001
Positive	18 (75.0%)	49 (48.5%)	24 (28.2%)	
Antiretroviral therapy use				
Yes	9 (45%)	20 (40%)	10 (42%)	0.930
No	11 (55%)	30 (60%)	14 (58%)	
Night sweats				
No	8 (33.3%)	29 (28.7%)	18 (21.2%)	0.350
Yes	16 (66.7%)	72 (71.3%)	67 (78.8%)	
Weight loss				
No	4 (16.7%)	21 (20.8%)	13 (15.3%)	0.610
Yes	20 (83.3%)	80 (79.2%)	72 (84.7%)	
Bacillary load				
Very low	4 (18%)	12 (12%)	6 (7%)	0.091
Low	6 (27%)	22 (22%)	11 (13%)	
Medium	8 (36%)	38 (38%)	29 (35%)	
Very high	4 (18%)	27 (27%)	37 (45%)	
Body mass index				
Normal	7 (29.2%)	30 (29.7%)	33 (38.8%)	0.550
Underweight	16 (66.7%)	67 (66.3%)	51 (60.0%)	
Overweight	1 (4.2%)	4 (4.0%)	1 (1.2%)	
CD4 cell count				
Normal	4 (16.7%)	33 (32.7%)	51 (60.0%)	<0.001
<LLN	20 (83.3%)	68 (67.3%)	34 (40.0%)	
CD8 cell count				
Normal	12 (50.0%)	66 (65.3%)	62 (72.9%)	0.100
<LLN	12 (50.0%)	35 (34.7%)	23 (27.1%)	
CD4:CD8 ratio				
Normal	5 (20.8%)	57 (56.4%)	66 (77.6%)	<0.001
<LLN	19 (79.2%)	44 (43.6%)	19 (22.4%)	
Mean corpuscular volume				
Normocytic	11 (45.8%)	42 (41.6%)	41 (48.2%)	0.660
Microcytic	13 (54.2%)	59 (58.4%)	44 (51.8%)	
Mean corpuscular haemoglobin				
Normochromic	16 (66.7%)	51 (50.5%)	57 (67.1%)	0.053
Hypochromic	8 (33.3%)	50 (49.5%)	28 (32.9%)	

LLN, lower limit of normal for Ugandans (normal ranges for CD4 and CD8 counts and CD4:CD8 ratio were 418–2105 cells per microliter (μl), 256–1619 cells/μl, and 0.52–4.1, respectively [47]).

### Factors independently associated with anaemia among people with TB in Uganda

As shown in Table 3, at multivariate analysis, anaemia was associated with being underweight (odds ratio (OR) 2.93, 95% CI 1.70–5.05, *P* < 0.01), low CD4:CD8 ratio (OR 2.54, 95% CI 1.07–6.04, *P* = 0.035) and hypochromia (OR 4.23, 95% CI 2.17–8.25, *P* < 0.01).

**Table 3.** Multivariate logistic regression model for factors associated with anaemia among people with TB in Uganda

Characteristic	Odds ratio	[95% Conf interval]		*P* value
Education level				
Secondary/tertiary	1			
None/primary	1.08	0.62	1.88	0.774
History of tobacco use				
Never smoked	1	.	.	.
Ever smoked	1.83	0.98	3.43	0 0.060
Type of resident				
Urban	1	.	.	.
Rural	0.84	0.48	1.47	0.539
HIV status				
Negative	1	.	.	.
Positive	1.65	0.77	3.52	0.195
Night sweats				
No	1	.	.	.
Yes	1.10	0.61	1.98	0.762
Weight loss				
No	1	.	.	.
Yes	1.19	0.62	2.27	0.608
Bacillary load				
Very low	1	.	.	.
Low	0.78	0.31	1.93	0.589
Medium	1.48	0.63	3.52	0.371
Very high	1.94	0.77	4.90	0.159
Body mass index				
Normal	1	.	.	.
Underweight	2.93	1.70	5.05	<0.001
Overweight	0.74	0.20	2.73	0.651
CD4 cell count				
Normal	1	.	.	.
<LLN	1.88	0.94	3.75	0.075
CD8 cell count				
Normal	1	.	.	.
<LLN	1.78	0.93	3.41	0.084
CD4:CD8 ratio				
Normal	1	.	.	.
<LLN	2.54	1.07	6.04	0.035
Mean corpuscular volume				
76–96 (femtolitres)	1	.	.	.
<76 femtolitres	1.31	0.75	2.28	0.351
Mean corpuscular haemoglobin				
Normal	1	.	.	.
Hypochromia	4.23	2.17	8.25	<0.001

LLN, lower limit of normal for Ugandans (normal ranges for CD4 and CD8 counts and CD4:CD8 ratio were 418–2105 cells per microliter (μl), 256–1619 cells/μl and 0.52–4.1, respectively [47]).

## Discussion

In this study, we evaluated the prevalence and factors associated with anaemia among people with TB in Uganda. Almost 60% of people with TB had anaemia, 60% of whom had moderate or severe anaemia. This result is in agreement with the global estimate of the prevalence of anaemia in TB of 62% [5]. The high proportion of TB patients with anaemia should be of public interest. Anaemia adversely affects TB outcomes in several ways. First, data from the general population suggest that anaemia is associated with all-cause mortality independent of age, sex and cardiovascular disease [23]. It is therefore not surprising that anaemia (with or without iron deficiency) has been associated with a threefold risk of mortality among people with TB [9]. Second, anaemia is emerging as an important risk factor for TB infection and disease in HIV-negative and HIV-positive individuals in a dose-dependent manner, regardless of the type of anaemia [4, 24, 25]. Lastly, anaemia in TB and the associated systemic inflammation do not invariably resolve on TB therapy, and could pose risk for other complications even after TB cure [6, 26, 27]. Therefore, there is the need for intensification of population-wide interventions to reduce the burden of anaemia in TB and HIV high-burdened countries as part of the strategies to reduce the incidence of TB. Additionally, guidelines for the management of anaemia in TB are needed to improve TB outcomes in patients with anaemia. Also, prospective studies are desirable to further characterise the evolution of anaemia and its complications (if any) after TB cure.

In the present study, anaemia was associated with night sweats, a longer duration of fever, low BMI, hyperthermia, high sputum bacillary loads, HIV co-infection, and low CD4 and CD8 counts at bivariate analysis. These findings suggest that anaemia is associated with the features of severe TB disease. Low BMI, elevated temperature and night sweats are part of a well-validated TB severity score (the Bandim TBscore) [28]. Moreover, HIV co-infection with severe immune suppression and high baseline mycobacterial loads are established risk factors for mortality in drug-susceptible and drug-resistant TB [29, 30]. Subsequent studies should evaluate the utility of integrating haemoglobin levels in the existent TB severity scores since clinical evaluation of anaemia (pallor) does not correlate well with haemoglobin measurements [31, 32]. Similar to our findings, de Mendonça *et al*. [8] recently reported anaemia to be associated with severe TB, low BMI and HIV co-infection in Brazil. We did not find any association of anaemia with advanced age and female sex as was reported by Lee *et al*. [33] in South Korea. This could be because they had a relatively older population with a median age of 44 years (compared to the >60% of our population which was aged ≤35 years). It is unclear why more people without anaemia reported weight loss than people with anaemia in our study. However, this was through self-reports, and we did not objectively confirm the weight loss. Self-reports are therefore not very reliable.

The association of TB-related anaemia with low BMI, as observed in our multivariate analysis is consistently observed in literature [34, 35]. Malnutrition is likely to be the common cause for both anaemia and low BMI in TB [36]. It is also hypothesised that abnormalities in appetite mediators – leptin and ghrelin – and inflammatory cytokines in TB could concurrently cause low nutrient intake, iron trapping in the reticuloendothelial cells and alter fat metabolism [37, 38]. The association of anaemia with a low CD4:CD8 ratio, independent of HIV infection, is interesting. A low CD4:CD8 ratio has been associated with altered immune responses, immune senescence and chronic inflammation and may predict mortality and morbidity in individuals with and without HIV [39]. The role of the CD4:CD8 ratio is not well established in TB. One meta-analysis has shown that newly diagnosed TB patients have reduced CD4:CD8 ratios compared to normal controls [40]. The CD4:CD8 ratio may also predict TB drug resistance [41, 42]. The relationship between the CD4:CD8 ratio and anaemia has been evaluated mostly in the context of iron deficiency. Iron deficiency anaemia is associated with a low CD4:CD8 ratio which improves on iron supplementation in children [43–45]. Iron is essential for proliferation and activation of CD4+ helper T-lymphocytes and intracellular iron deficiency could impair the function of the enzymes that drive the metabolic and redox reactions involved in these processes [46]. Therefore, our finding suggests iron deficiency as a possible cause of low CD4 counts (hence low CD4:CD8 ratio) and this seems to occur in a dose-dependent manner since more patients with severe anaemia had low CD4 T-cell counts and CD4:CD8 ratios than those with a mild form. This relationship between anaemia severity and T-cell counts was not observed with CD8 cell counts.

From our study, it is difficult to delineate the cause of anaemia. Microcytosis and hypochromia were observed to be a commoner among patients with anaemia as has been shown elsewhere [8, 33, 35]. This could suggest anaemia of chronic disease or iron deficiency which account for 50% and 20% of anaemia in TB, respectively [5]. However, nutritional causes, such as iron deficiency, are likely in our study population as evidenced by the association of anaemia with low BMI and low CD4:CD8 ratio as discussed above. The cross-sectional nature of our study is unable to establish the direction of the relationship between anaemia and TB; that is, whether anaemia was a risk factor for TB or TB was the cause of anaemia. Considering the relatively short duration of most symptoms (≤30 days), it is reasonable to suggest that anaemia preceded clinical TB disease. However, this is best ascertained by prospective studies.

Our study has limitations. First, we could not evaluate the association between anaemia and radiological manifestations because the data were not available. This would otherwise better characterise TB severity. Second, confounders such as helminth co-infection, gastrointestinal bleeding and dietary habits were not evaluated for. Nevertheless, we were able to assess for association with HIV, haemoparasites (malaria) and drugs (ART and cotrimoxazole). Another limitation is that the study was conducted at a referral facility and the results could be affected by referral bias, in which case patients with severe TB and/or anaemia could have been preferentially referred for care at the NTTC. This limits the generalisability of our study. However, at the NTTC, <30% of patients are referral cases, and a minority of patients (28%) were from rural areas (presumed to have been referred). Additionally, the hospitalisation status and history of previous transfusions among the participants were not documented, which could be confounders. Moreover, some variables such as the clinical symptoms and symptom duration were self-reported and could be affected by recall bias. Lastly, data on iron studies, serum hepicidin and cytokines were not available to enable us to type the anaemia.

## Conclusion

The prevalence of anaemia was high among people with bacteriologically confirmed TB at the NTTC in Uganda. Majority of the patients have moderate to severe anaemia. Anaemia was associated with the features suggestive of severe TB disease: high bacillary load, night sweats, low BMI, longer duration of fever, hyperthermia, HIV co-infection, low T-cell counts and low CD4:CD8 ratio. There is the need to include anaemia in TB severity scores. Guidelines for managing anaemia in TB are needed in low-income settings where facilities for establishing specific aetiology of the anaemia are not readily available.

## Data Availability

Datasets used in this analysis are available from the corresponding author upon reasonable request.
